# Alternative classifications of neurons based on physiological properties and synaptic responses, a computational study

**DOI:** 10.1038/s41598-019-49197-8

**Published:** 2019-09-11

**Authors:** Ferenc Hernáth, Katalin Schlett, Attila Szücs

**Affiliations:** 1 MTA-ELTE-NAP B Neuronal Cell Biology Research Group, Eötvös Loránd University, Budapest, Hungary; 2 BioCircuits Institute, University of California San Diego, La Jolla, California United States; 3 Balaton Limnological Institute of the Center for Ecological Research, Hungarian Academy of Sciences, Tihany, Hungary

**Keywords:** Computational models, Biophysical models

## Abstract

One of the central goals of today’s neuroscience is to achieve the conceivably most accurate classification of neuron types in the mammalian brain. As part of this research effort, electrophysiologists commonly utilize current clamp techniques to gain a detailed characterization of the neurons’ physiological properties. While this approach has been useful, it is not well understood whether neurons that share physiological properties of a particular phenotype would also operate consistently under the action of natural synaptic inputs. We approached this problem by simulating a biophysically diverse population of model neurons based on 3 generic phenotypes. We exposed the model neurons to two types of stimulation to investigate their voltage responses under conventional current step protocols and under simulated synaptic bombardment. We extracted standard physiological parameters from the voltage responses elicited by current step stimulation and spike arrival times descriptive of the model’s firing behavior under synaptic inputs. The biophysical phenotypes could be reliably identified using classification based on the ‘static’ physiological properties, but not the interspike interval-based parameters. However, the model neurons associated with the biophysically different phenotypes retained cell type specific features in the fine structure of their spike responses that allowed their accurate classification.

## Introduction

All the intricate molecular machinery that neurons engage in their operation serves a principal goal: converting synaptic inputs into action potentials, the fundamental events of information transfer in the brain. This process involves the complex interplay of multiple voltage-gated membrane currents. Electrophysiologists commonly utilize current step stimulation to elicit subthreshold voltage responses and firing to extract the neurons’ physiological parameters. This approach, while technically simple, has been very successful in identifying and classifying a variety of neuron types^1–4^. The firing pattern, elicited by suprathreshold current injection, is commonly referred to as the intrinsic firing pattern of the neuron^5^. According to this scheme, neurons are classified into subtypes such as regular firing, spike-frequency adapting, bursting types, among others^6,7^. Here, an important question arises: do neurons categorized into these well-established physiological types keep their similarity in their firing responses under the action of real synaptic inputs? One has to recognize that neurons, during their normal operation, receive a mixture of rapidly fluctuating excitatory and inhibitory synaptic conductances^8^. It is technically challenging to accurately measure the physiological parameters of neurons in intact brain circuits and concurrently analyze their synaptic responses. Hence, one can only assume that neurons that appear as belonging to the same physiological phenotype will transform real synaptic inputs into consistent output patterns. This idea could be assessed by exposing biophysically well characterized neurons to identical spatio-temporal patterns of synaptic input, a requirement hard to meet in complex neuronal circuits. However, one can take advantage of synthetic synaptic inputs and the dynamic clamp technique^8,9^ to analyze firing responses of biophysically different neuronal phenotypes. Indeed, our experiments with neurons of the rat extended amygdala demonstrated that even biophysically different neurons can transform simulated synaptic inputs into qualitatively similar firing patterns^10^. As an alternative to biological experiments, computational modeling can be a convenient approach to this problem and this is what we employ in the present study. We create a diverse population of model neurons based on three generic phenotypes and mimic current clamp experiments to investigate the neurons’ observable physiological parameters. Next, we expose the same set of model neurons to simulated synaptic bombardment and record their firing responses. We show that classification schemes based on the static current step responses and the neuron’s firing under synaptic inputs are not necessarily equivalent. Additionally, supervised and unsupervised classification methods produce output that allows alternative interpretations of the physiological diversity of neuron populations.

## Results

Our strategy was to generate a database of biophysically diverse population of model neurons representing three generic phenotypes and to analyze the neurons’ responses under different stimulus conditions. In the first set of simulations we aimed to measure various physiological parameters of the model neurons by stimulating them with rectangular current pulses. These physiological parameters were then used to differentiate between the neuron types using supervised and unsupervised classification methods. Next, we elicited firing responses of the same model neurons under simulated synaptic inputs and extracted spike arrival time-based parameters for classification.

### Construction of a biophysically diverse population of model neurons

**Figure 1:**
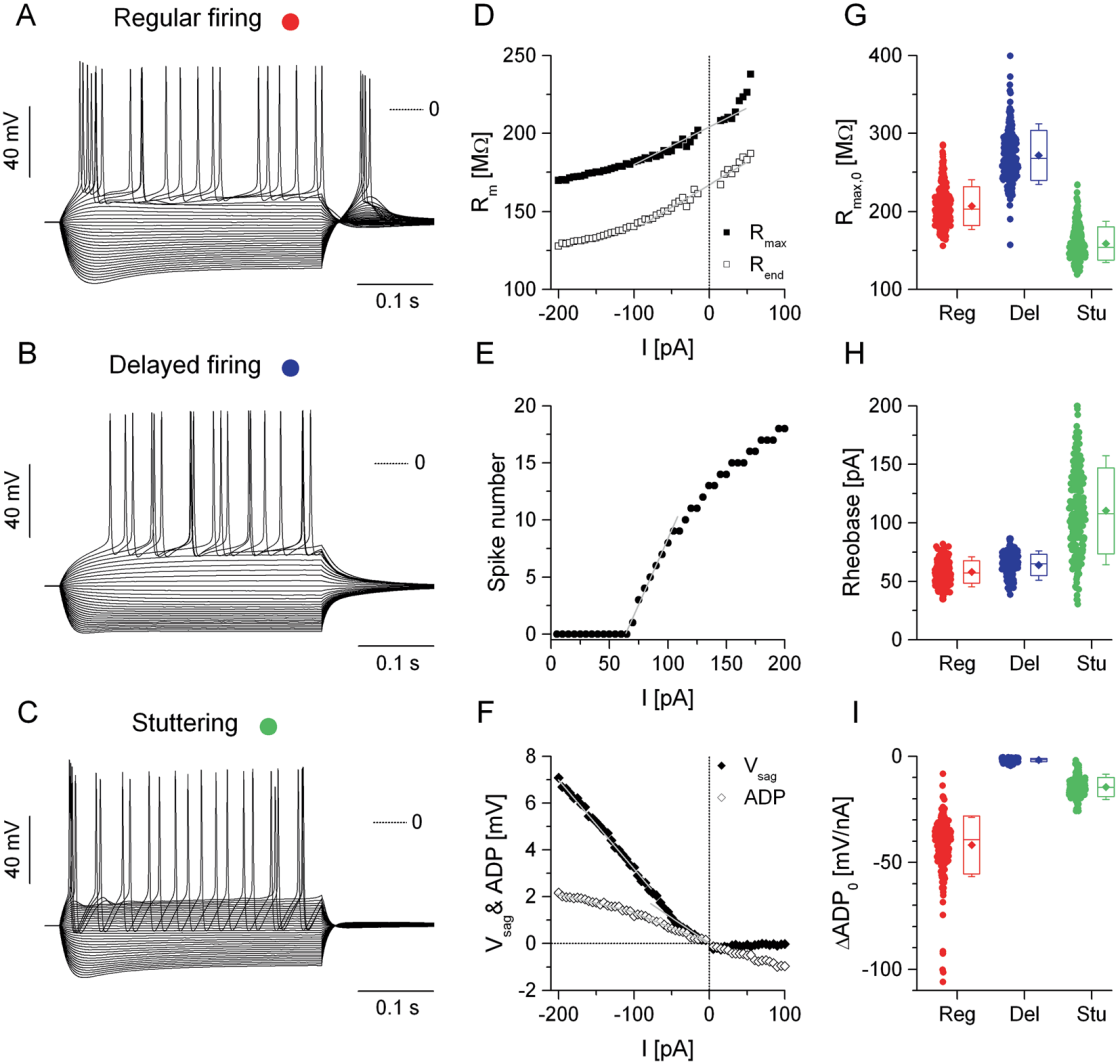
Three types of biophysically different model neurons produce distinctive voltage responses under current step stimulation. The voltage traces on the left are elicited using 400 ms current steps starting at −200 pA and incremented by 5 pA (**A**–**C**). Middle panels illustrate various procedures to analyze the model neurons’ physiological properties. (**D**) Membrane resistance is plotted against the injected current. (**E**) Spike number vs. current relationship. (**F**) voltage sag and afterdepolarization. Rightmost panels show selected physiological parameters for the three types of model neurons, 200 instances in each group (**G**–**H**). (**G**) Input resistance (R_max_ extrapolated to I = 0). (**H**) Rheobase. (**I**) The slope of the afterdepolarization function. Red, blue and green colors correspond to the regular firing, delayed firing and stuttering type model neurons as shown in (**A**–**C**).

We created a total of 600 model implementations based on 3 biophysical phenotypes^11^. These were based on our prior computational models of 2 types of rat bed nucleus of stria terminalis (BNST) neurons^11–13^ and a third model that was more representative of fast spiking/stuttering neurons^14^. The model neurons have dendritic, somatic and axonic compartments and the voltage-gated currents are distributed among the 3 compartments in a non-uniform manner. The voltage responses of these three generic model neurons under standard current step stimulation are shown in Fig. 1. The first model, referred to as the regular firing neuron exhibits a characteristic voltage sag under stimulation with hyperpolarizing current steps (Fig. 1A). This well-known behavior is mediated by the intrinsic h-current that activates at hyperpolarized membrane potentials. Additionally, post-inhibitory spikes are fired following the termination of negative current steps that is primarily mediated by the low-threshold Ca-current (I_T_) and also augmented by the h-current^15^. The second model phenotype exhibits strong inward rectification (Fig. 1B), i.e. decreased membrane resistance at hyperpolarized membrane potentials, due to the action of its inward rectifying K-current (I_Kir_)^12,16^. This voltage-dependent membrane conductance slowly deactivates when the membrane potential depolarizes under positive current steps and produces a characteristic voltage ramp preceding the first action potential^17^. This phenotype is referred to as the delayed firing type neuron. The firing pattern of the third model neuron is markedly different from that of the previous phenotypes (Fig. 1C). Spikes here are followed by strong afterhyperpolarization (AHP) and the interspike intervals exhibit irregular ‘jumps’ as the current level is gradually increased near rheobase levels. This type of firing pattern is often referred to as stuttering and it is mainly determined by the neurons’ intrinsic K-current that activates and inactivates slowly (K_slow_)^14^. Firing at higher levels of depolarizing current becomes more regular and the model exhibits low variability in the interspike intervals.

The voltage responses shown in Fig. 1 are analogous to those electrophysiologists commonly record in whole-cell patch clamp experiments. Procedures to extract some of the physiological parameters are demonstrated in Fig. 1D–F (also described in^16^). Based on the 3 generic neuron models we built a biophysically diverse population of model neurons by randomly varying the maximal conductance of the intrinsic membrane currents and the passive membrane parameters. Maximal conductance of each varied voltage-gated current was drawn from a Gaussian distribution centered at the value of the generic model and having 40% standard deviation. The somatic leakage conductance and membrane capacitance parameters were also varied using 25% standard deviation. We produced 200 implementations for each phenotype and stimulated the model neurons using the current step protocol as shown in Fig. 1. Hence, we obtained physiological parameters of a total of 600 model neurons. Figure 1G–I demonstrate the spread of 3 selected physiological parameters calculated from the current step responses. We used a total of 20 parameters, referred to as static, or IV features, for the subsequent clustering and classification study (Table 1). The 20 physiological parameters provided a detailed description of the dynamical behavior of the model neurons under current step stimulation.

**Table 1:** Physiological parameters used to construct IV feature vectors.

Parameter	Description
V_r_	Resting membrane potential.
R_max,0_	Membrane resistance extrapolated to I = 0 by linear fitting. Peak voltage deflections are used to calculate individual resistance values.
ΔR_max,0_	The slope of the linear fit of the R_max_ vs. current curve near I = 0.
R_end,0_	Membrane resistance extrapolated to I = 0 by linear fitting. Voltage deflections at the end of the stimulus step are used to calculate resistance values.
ΔR_end,0_	The slope of the linear fit of the R_end_ vs. current curve near I = 0.
ΔV_sag,0_	The slope of the linear fit of the voltage sag vs. current relationship near I = 0.
ΔV_sag,hyp_	The slope of the linear fit of the voltage sag vs. current relationship at current levels negative to the midpoint (below −60 pA).
ΔV_sag_ ratio	Ratio of the above 2 voltage sag slope parameters.
ΔADP_0_	The slope of the linear fit of the afterdepolarization vs. current relationship near I = 0.
ΔADP_hyp_	The slope of the linear fit of the afterdepolarization vs. current relationship at current levels negative to the midpoint (below −70 pA).
ΔADP ratio	Ratio of the above 2 afterdepolarization slope parameters.
Rel. V_sag,0_	Mean relative voltage sag (expressed as percentage) between I = 0 and the midpoint current level.
Rel. V_sag,hyp_	Mean relative voltage sag (expressed as percentage) between the midpoint current level and −200 pA.
Rel. ADP_0_	Mean relative afterdepolarization (expressed as percentage) between I = 0 and the midpoint current level.
Rel. ADP_hyp_	Mean relative afterdepolarization (expressed as percentage) between the midpoint current level and −200 pA.
Rheo.	Rheobase, the minimal level of depolarizing current eliciting an action potential.
ISI Acc.	Interspike interval accommodation index.
ISI C.V.	The coefficient of variation of interspike intervals.
Rel. PIR	Relative number of spikes fired under post-inhibitory rebound (after the termination of negative current steps).
ϕ_Vmin_	The mean phase of the location of minimal membrane potential between spikes.

### Correlation between the static features and biophysical properties

**Figure 2:**
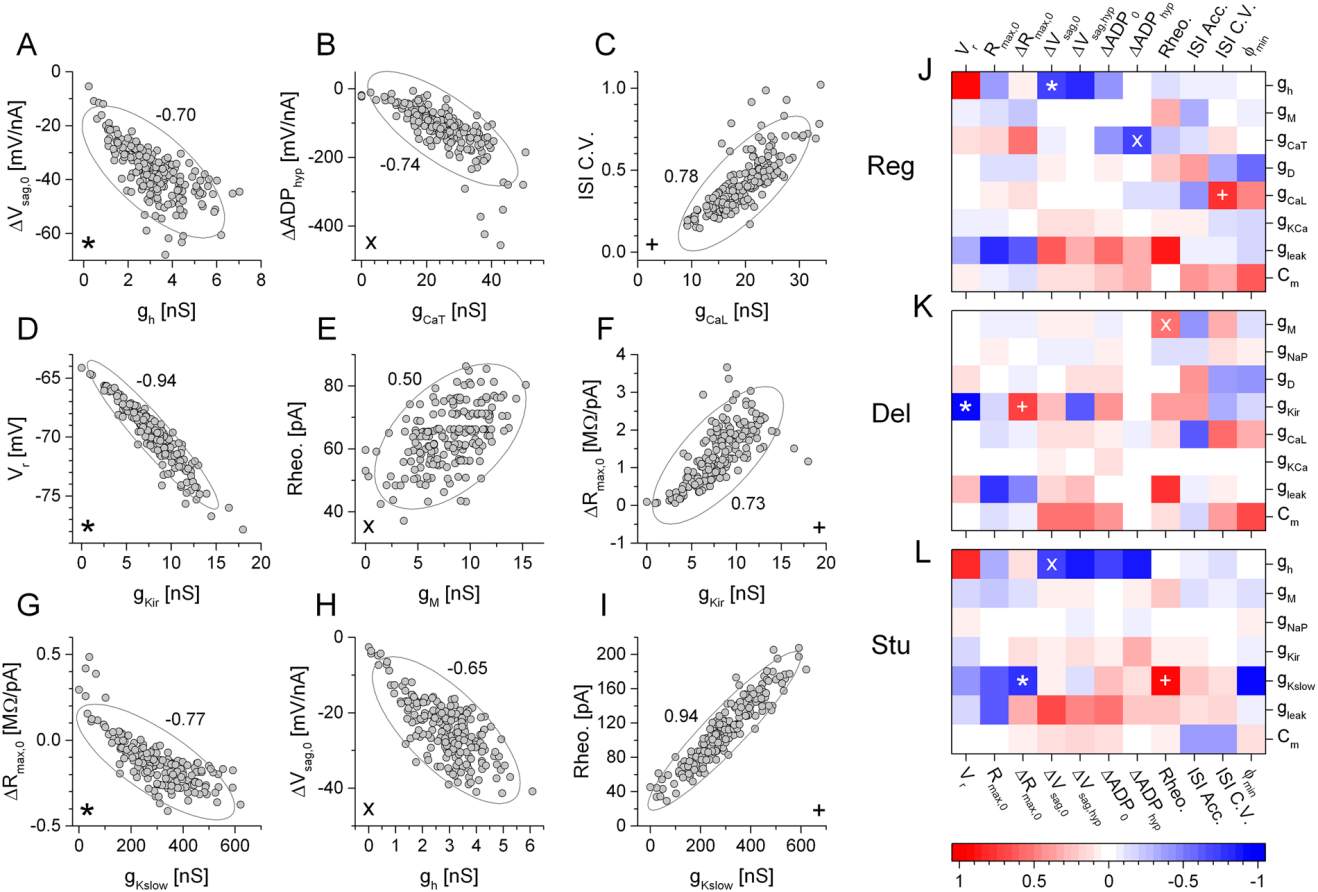
Maximal conductances of specific voltage-activated membrane currents and physiological parameters of the model neurons are correlated. (**A**–**I**) Selected physiological parameters are plotted against the maximal conductance of a voltage-gated current present in the regular firing (top row), delayed firing (middle) and stuttering (bottom) type models. Confidence ellipses appearing in each graph were obtained by calculating Pearson correlation (Pearson-coefficients are shown next to the ellipses). The h-current has a clear effect in setting the magnitude of voltage sag (**A,H**), while K-currents have stronger role in regulating the resting membrane potential (**D**), and rheobase (**E,I**) of the model neurons. Color maps on the right depict the degree of Pearson-correlation between the intrinsic voltage-gated currents and 11 selected physiological parameters obtained from the current step data. Asterisks, crosses and plus symbols in the color maps indicate the corresponding scatter plots in (**A**–**I**).

One reason electrophysiologists favor current step stimulation is because such experiments provide valuable information on the intrinsic biophysical properties of the neurons. Indeed, several features of the voltage responses, e.g. the first spike latency, depolarizing voltage sag indicate the action of specific intrinsic membrane currents. Knowing the maximal conductance values of each voltage-dependent current in our model simulations, we can directly evaluate the correlation between the measured IV features and the intrinsic biophysical parameters. Figure 2 shows examples of such correlations for the three neuronal phenotypes. As indicated above, the h-current is primarily responsible for setting the magnitude of the voltage sag that develops under stimulation with hyperpolarizing current steps and pharmacological blocking of the h-current reliably eliminates the voltage sag^18^. Indeed, we find a strong correlation between g_h_ and the slope of the voltage sag curve (ΔV_sag,0_; Fig. 2A,H). As another example, the rheobase of the delayed firing model tends to increase with the magnitude of the M-current (Fig. 2E) while the resting membrane potential is mainly influenced by its inward rectifying K-current (Fig. 2D). These examples clearly show that data from current clamp experiments provide valuable insight into the biophysical properties of neurons and one can, at least comparatively, assess the magnitude of specific voltage-gated currents in shaping the neurons’ dynamical behavior.

A more systematic analysis of the relation between observed physiological parameters and the strength of intrinsic membrane currents is demonstrated in Fig. 2J–L. We calculated Pearson-correlation values for the most informative 11 IV features and the varied intrinsic parameters of the models. These included the maximal conductances of voltage-gated currents, the leakage conductance and the membrane capacitance. Clearly, the impact of intrinsic biophysical properties in shaping the physiological behavior of the model depends on the cell type. As an example, an increase of the membrane capacitance will result in a positive shift of the interspike interval variability (ISI C.V.) in the regular and delayed type models but has the opposite effect in the stuttering type neurons. The strong correlation between the intrinsic voltage-gated currents and the IV features suggest that multivariate analysis of these parameters can effectively differentiate the biophysically different neuron types. Indeed, even in 2-dimensional scatter plots of selected physiological parameters shown for the 600 model implementations we can recognize the separation of clusters representing the 3 biophysical phenotypes (Fig. S1 in Supplementary information).

### Multivariate analysis and classification of neurons based on IV features

**Figure 3:**
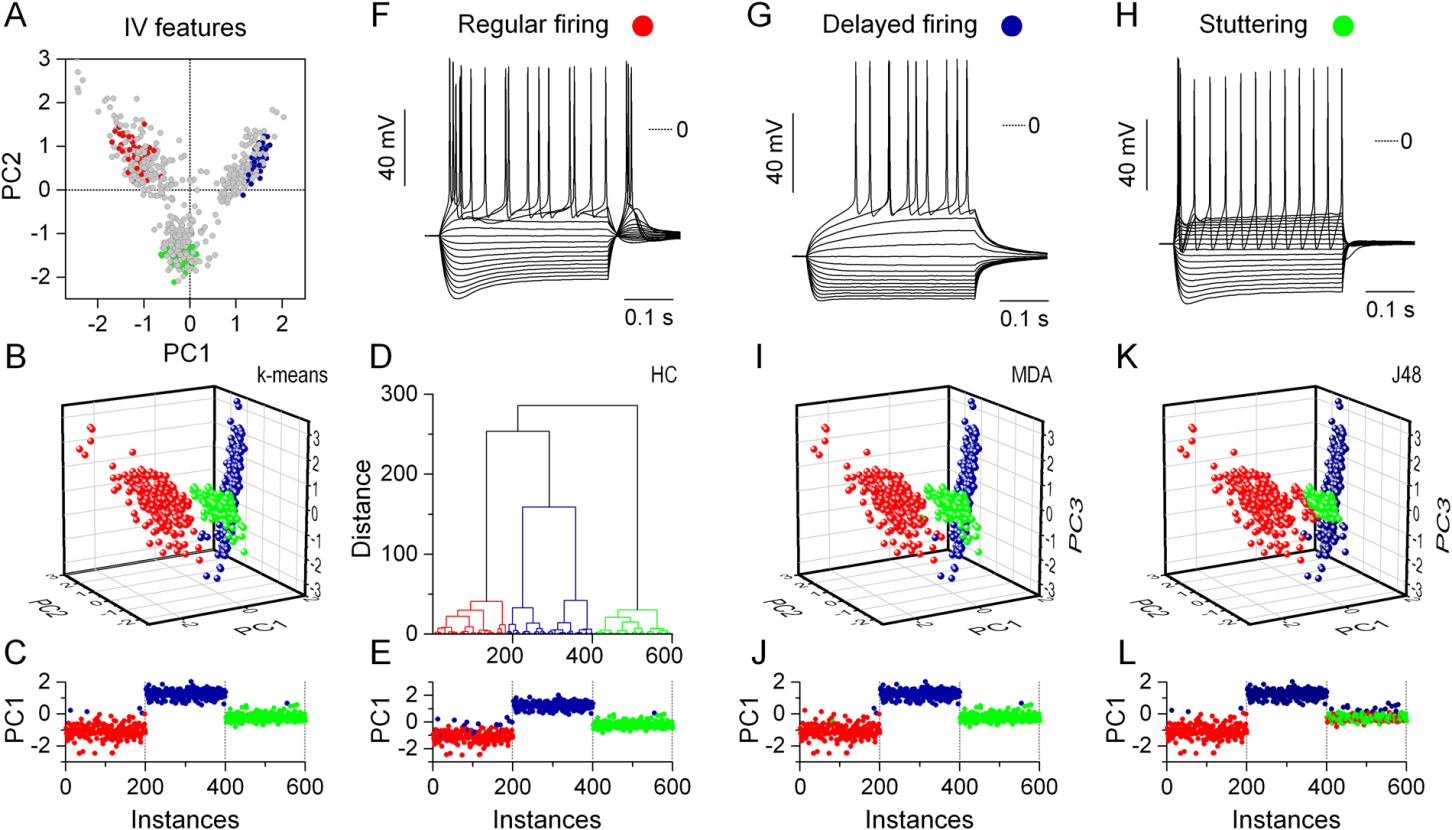
Classification algorithms using physiological parameters identify three groups of neurons corresponding to the three biophysical phenotypes. The first two principal components calculated from the 20-dimensional IV feature vectors are scatter plotted in (**A**). For unsupervised clustering we use 3-dimensional PC vectors and color them according to the membership values determined by the k-means (**B**) and hierarchical clustering (**D**) methods. PC1 vs. instance number plots demonstrate how accurately these methods identify the different biophysical phenotypes. (**C,E**) Here, the first 200 instances are all regular firing type cells followed by the delayed and stuttering types. As training set for the multidiscriminant analysis (MDA) and J48 methods we manually selected a total of 120 (3 × 40) model instances and labeled them as regular firing, delayed firing and stuttering neurons (colored points in **A**). Representative examples of the voltage responses of such model neurons are shown in (**F–H**). The output of the MDA and J48 methods based on this training set are shown in (**I**–**L**). Both supervised classification methods identify the original 3 groups of biophysical phenotypes almost perfectly.

Prior to performing unsupervised clustering of the data we used principal component analysis (PCA) to reduce correlations between static physiological parameters and to aid the visualization of the results from the clustering^6,19^. The first 3 principal components (PCs) accounted for 86% of total variability in the entire dataset (600 implementations), allowing the use of the 3-dimensional, PCA-transformed vectors for cluster analysis. Next, we ran 2 unsupervised clustering algorithms on the PCA-transformed vectors. The hierarchical- (HC) and k-means clustering methods have been commonly used by electrophysiologists to investigate functional diversity of neuronal populations^1–3^. To evaluate the performance of these algorithms we compared the calculated cluster membership data to the known phenotype labels that were readily available from the model simulations. By plotting the first principal component of all 600 model implementations as a sequence of points and using the colors representing the cluster membership we can quickly assess the goodness of clustering (Fig. 3C). Here, the first 200 points belong to the regular firing phenotype, followed by the delayed firing and stuttering models. Correct identification of cluster membership would yield 3 subsequent sets of 200 points colored as red, blue and green. Indeed, as shown for the k-means method, we find a near perfect match (99.2%) between the membership data and the phenotype labels with only a few cells incorrectly identified as belonging to the delayed firing phenotype (sparse blue points below 200, Fig. 3C). Similarly, the HC algorithm performs well in separating the data into 3 phenotypes (Fig. 3D,E).

The overall performance of the 2 unsupervised methods is compelling. At the same time, supervised classification algorithms and machine learning offer a powerful alternative to such methods. Indeed, expertise of researchers who routinely analyze electrophysiological data from biological neurons continues to be a valuable input to train classifiers. Therefore, in the next phase we used two supervised classification methods, namely the multiple discrimination analysis (MDA)^1,20^ and the J48 (also referred to as C4.5)^21^ decision tree algorithms. As training set to these methods, we selected 3-times 40 model instances that produced voltage responses we considered as representative examples of the underlying biophysical phenotype. The visual assessment of the models was based on voltage traces such as in Fig. 3F–H. For each phenotype, we labeled 40 of such instances (labeled cells appear in color in Fig. 3A). As shown in Fig. 3I,J, the discrimination method performed superbly and only 4 of the 600 model instances were not correctly identified by it. The J48 algorithm worked somewhat less accurately, because a higher percentage of stuttering neurons were assigned to the wrong phenotype (Fig. 3K,L). In conclusion, both supervised and unsupervised classification methods perform very well in discriminating among biophysically different model neurons when physiological parameters extracted from current step responses are used as input.

### Classification based on interspike interval data

**Figure 4:**
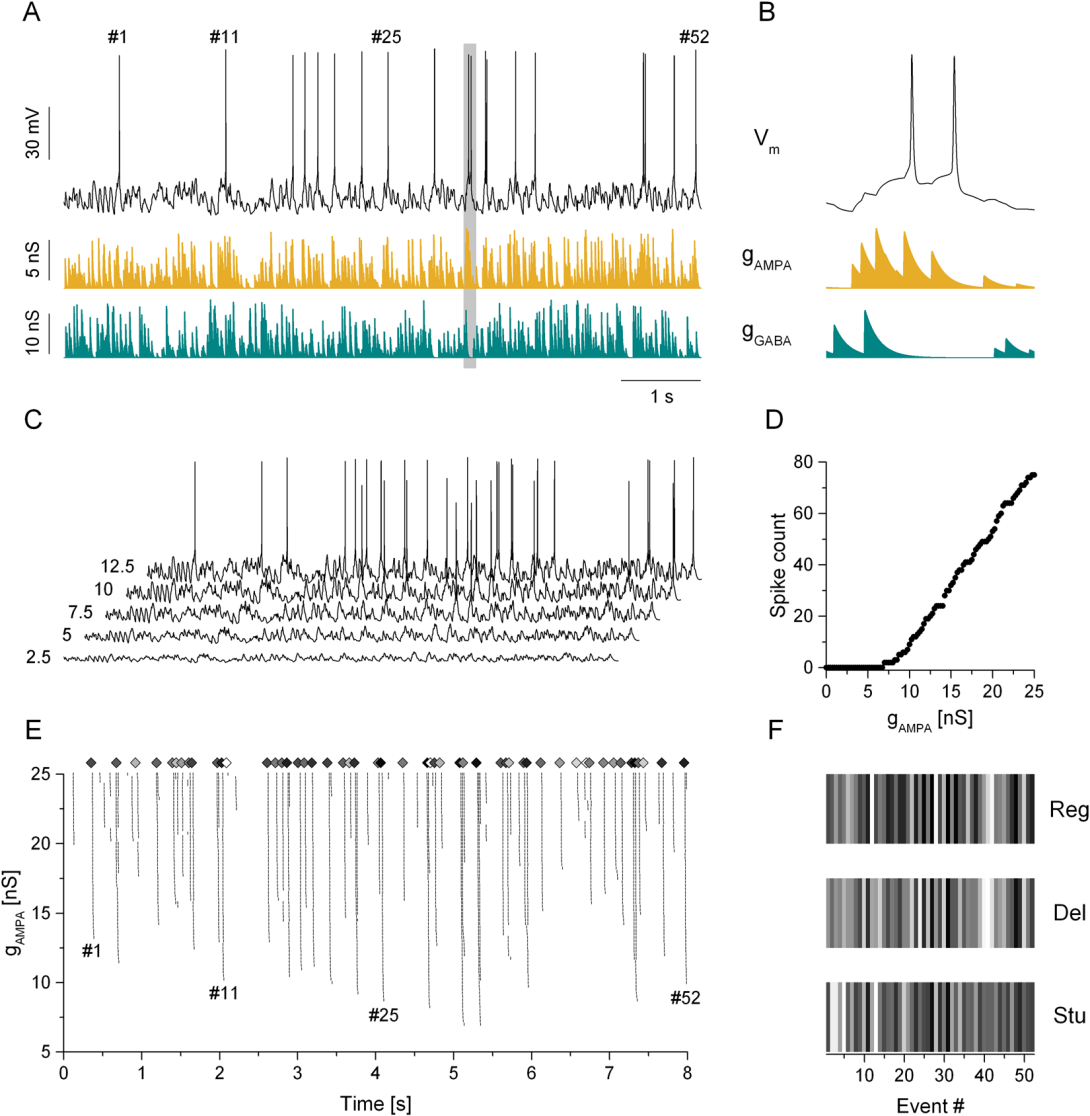
The firing responses of model neurons under simulated synaptic bombardment allow the construction of spike event vectors for clustering and classification. Panel A shows an 8-s length voltage trace (black) of a regular firing type model neuron under the action of simultaneous excitatory (gold trace) and inhibitory (cyan) inputs. The shaded section in (**A**) is enlarged in. (**B**) Voltage traces under gradually stronger excitatory/inhibitory inputs exhibit an increasing number of spikes (**C**). Numbers on the left of the traces indicate the AMPA conductance level used for the corresponding sweep. (**D**) The input-output relationship obtained from such protocol. The raster plot in (**E**) demonstrates the firing pattern of the model neuron stimulated under such stimulation. Here, arrival times of spikes are plotted on the X-axis while the excitatory synaptic conductance are plotted on the Y-axis. Diamond symbols indicate 52 event locations where firing probability is found to be positive for most of the model neurons. Panel **F** demonstrates the firing reliability in the 52 event locations for the three biophysical phenotypes. These event vectors, shown as grayscale ‘barcodes’ here, are calculated for all model implementations and are used as input data for classification.

**Figure 5:**
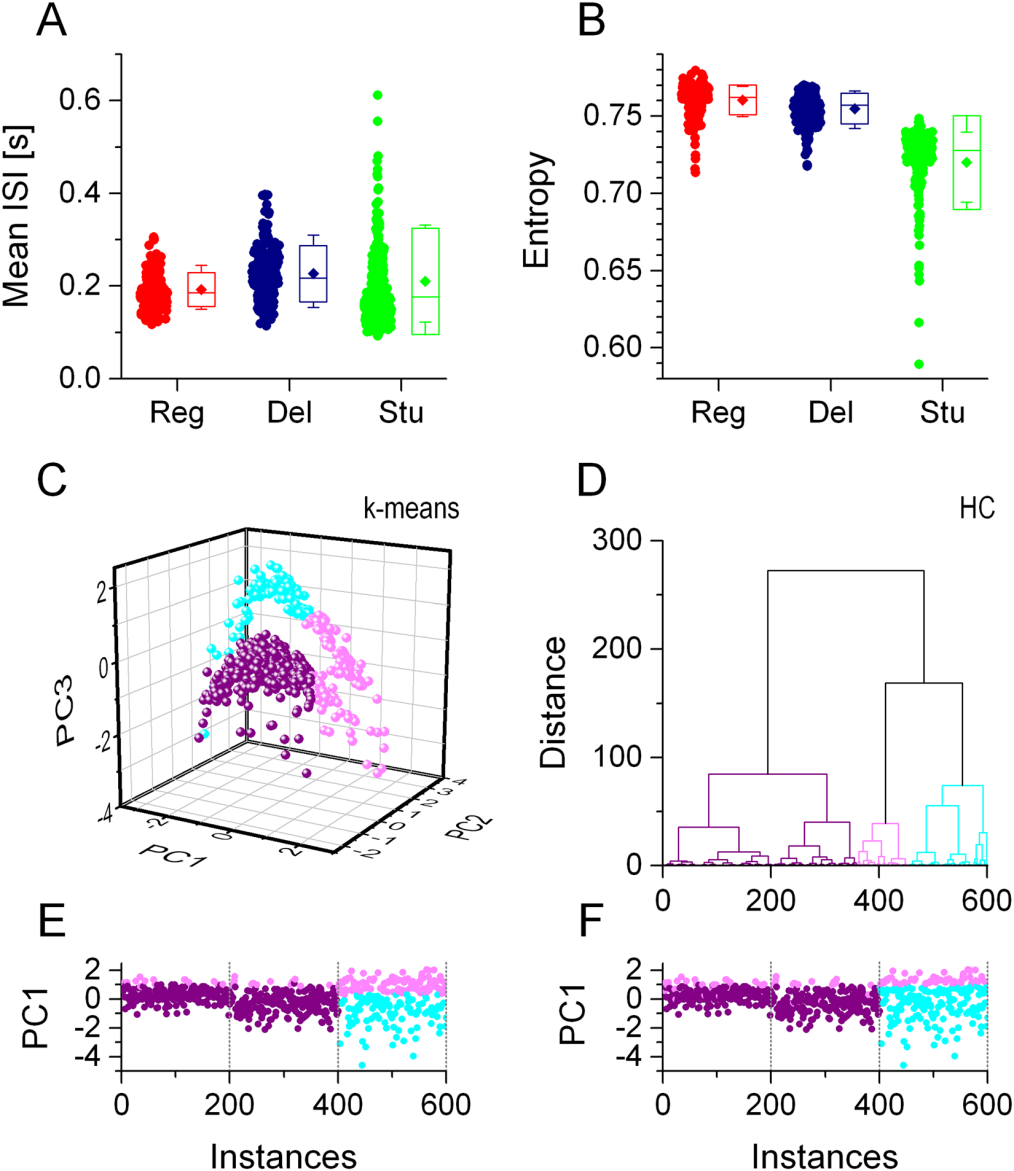
K-means and hierarchical clustering of neurons based on their interspike interval statistical parameters does not reproduce the distribution of the 3 biophysical phenotypes. Mean interspike interval data are shown for the 3 biophysical phenotypes in (**A**). The different model implementations exhibit a high variance in the mean ISI values and populations corresponding to the 3 groups largely overlap. (**B**) ISI-entropy values calculated from the firing responses shown for the 3 phenotypes. Stuttering type models exhibit lower entropy in the firing responses. (**C**) 3-dimensional plot of PC vectors clustered by the k-means algorithm. The majority of vectors from instances 1 to 400 are grouped together and represented by purple colored symbols (**E**). Hierarchical clustering of the same data yields a similar distribution (**D,F**).

In contrast to physiological parameters observed under current step stimulation electrophysiologists often rely on spike time data acquired extracellularly. Here, temporal parameters of firing patterns are analyzed to reveal potential cell type-specific features. Our next simulations focused on how the population of 600 model neurons can be classified based on firing responses these neurons produce under simulated synaptic bombardment. We fed into the model neurons a mixture of excitatory and inhibitory synaptic inputs that resulted in fluctuating voltage traces and action potentials appearing in an irregular pattern (Fig. 4A). The duration of the two uncorrelated, Poissonian-type waveforms was 8 s. To characterize the input-output transformation of the models in such scenario, we incremented the synaptic conductance of the AMPA-connection from 0 to 25 nS in 0.25 nS steps and set the strength of the GABA-connection twice of that of the AMPA^11^. The increasing amount of net excitation resulted in firing responses with an increasing number of spikes per sweep. This protocol yielded input-output functions (Fig. 4D) analogous to those under static current stimulation (Fig. 1E). Arrival times of spikes emitted during the entire synaptic stimulus protocol were recorded and used to extract interspike-interval based features for classification. We calculated 7 temporal parameters of the firing responses including the mean firing rate, total number of spikes, standard deviation and entropy of the interspike interval (ISI) distributions. It is important to recognize that these parameters preserve no information on the temporal order of spikes, rather, they characterize the overall intensity and variability of the responses. Two of these ISI-based parameters are demonstrated for the 3 biophysical phenotypes in Fig. 5A,B. In general, ISI-based parameters exhibited a great variability across model implementations and phenotypes. Next, we ran PCA and unsupervised clustering of these ISI-based vectors similarly to that performed on the IV features. K-means clustering of these vectors resulted in the class membership distributions shown in Fig. 5C,E. Hierarchical clustering reproduced the same overall distribution of data, as shown by the PC1 vs. instance number plot (Fig. 5F). Importantly, the two unsupervised algorithms suggest classes that do not match that of the biophysical phenotypes. In particular, purple colored datapoints, representing cluster #1, appear in a wide range, from instances 1 to 400, where the regular and delayed firing type neurons are listed. Besides, stuttering type model neurons occupying the range between 401 and 600, are assigned to 2 clusters. Accordingly, we display these class membership data in a color code that is different from the one we used to distinguish the biophysical phenotypes. These findings indicate that interspike interval-based parameters extracted from firing responses are not appropriate to correctly separate biophysically different neuronal phenotypes.

### Classification based on spike timing reliability

The peri-stimulus spike raster plot is an effective way to demonstrate spike arrival times of neurons firing under cyclic stimulation (Fig. 4E). We calculated such raster plots for each model neuron receiving simulated synaptic inputs. Importantly, we found that the majority of spikes arrived in well-defined time locations (windows) irrespective of the biophysical properties of the model neurons^10^. There were a relatively low number of possible temporal locations (spike events) where spike emission was probable. As shown in Fig. 4E, spikes tend to concentrate in narrow bands parallel to the conductance axis. This characteristics of the responses allowed us to construct reasonably low-dimensional feature vectors that were still informative in describing the firing response of the model neurons. We selected 52 spike event locations and calculated the reliability of firing for each location. Reliability was defined as the percentage of trial numbers when a postsynaptic spike was triggered in the particular event location. Plotting the 52 successive reliability values for three generic biophysical model phenotypes produces the graphs on Fig. 4F. These grayscale plots resembling barcodes are visual representations of the feature vectors we used to classify the neurons. It is important to note that these vectors, as ISI-based vectors above, are merely based on spike arrival data and they are not directly related to the physiological properties of the neuron.

**Figure 6:**
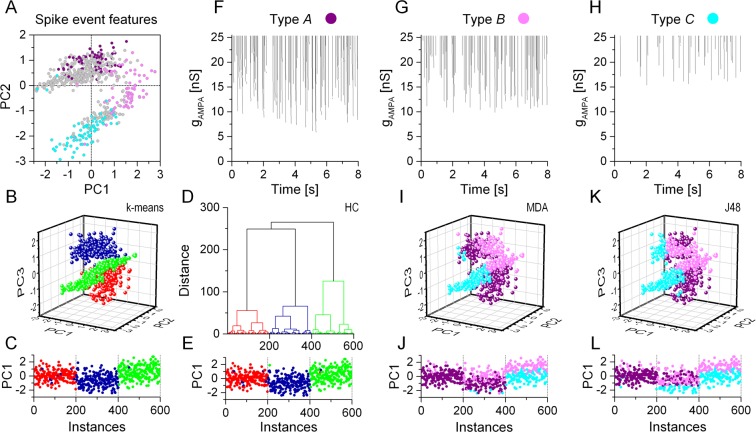
Unsupervised clustering algorithms perform excellently in identifying the 3 biophysical phenotypes using spike event reliability data. (**A**) Scatter plot of the first 2 PCs exhibit diffuse clusters of datapoints without indicating three distinct classes. The k-means (**B**), and hierarchical clustering (**D**) methods, however, correctly identify 3 separate groups of models corresponding to the original biophysical phenotypes (red, blue and green symbols). Sequential plots of the first PC as functions of the instance number are shown in (**C,E**). Visual inspection of the raster plots such as the ones in (**F**–**H**) suggests three qualitatively different firing patterns in the population of the 600 models. Correspondingly, we label model instances as belonging to one of the subjective type *A*, *B* and *C* classes. This set of labeled neurons (colored points in **A**) serves as the training set for the 2 classification methods. Results from the MDA and the J48 algorithms are shown in (**I**–**L**). The classification methods reproduce the general distribution of the training dataset and the clustered data based on the ISI-parameters (Fig. 5), but the 3 classes do not match the biophysical phenotypes.

The next question was whether the classification methods we used above would be able to uncover the three underlying biophysical phenotypes using these spike event reliability vectors. Prior to clustering we performed PCA to reduce dimensionality and to aid visualization of the spike event reliability data. The scatter plot of the first two principal components in Fig. 6A displays a more dispersed distribution of points than when plotting the PC vectors obtained from the IV features (Fig. 3A). The high degree of correlation between the event reliability features is shown by the fact that the first 2 PCs account for 80.2% of total variability and the third PC adds another 9.9% to that. Hence, we can use the first 3 PCs to create input vectors for the k-means and hierarchical clustering algorithms.

Surprisingly, the two unsupervised methods reveal the 3-cluster structure in the dataset representing the original biophysical phenotypes. Except in a few cases, cluster memberships are correctly determined by both the k-means and HC methods (Fig. 6B–E). These findings show that biophysically different types of model neurons integrate synaptic inputs in a way that produce firing patterns with cell type specific features. Hence, we find a strong contrast between the classification results from the ISI-based parameters vs. the spike event reliability vectors.

Next, we aimed to build a training dataset for the two supervised methods based on the spike event reliability data. Here, we attempted to detect qualitative differences in the raster plots without knowing the biophysical properties (phenotypes) of the model neurons and assign them to one of three subjective categories. In fact, visual evaluation of the peri-stimulus raster plots proved to be challenging. Model neurons in the first group, labeled as type *A*, produced firing patterns that exhibited a wide range of interspike intervals including both very short and very long ones (burstiness; Fig. 6F). We labeled n = 76 model instances as type *A*. The second group of cells, labeled as type *B*, produced more regular firing output (Fig. 6G, n = 79) and the distribution of the shorter ISIs was far more constrained than in the first group. The third group of model cells (type *C*) displayed no burstiness rather an abundance of long interspike intervals in the firing patterns (Fig. 6H, n = 74). Figure 6A shows the distribution of all 600 PC vectors including the labeled model instances according to our qualitative inspection (purple, magenta and cyan symbols). Since we found no good match between the labels from the visual assessment and the biophysical phenotypes, we used an alternative color scheme in the following panels (as in Fig. 5). Type *A* neurons (purple symbols) appear mostly among the first 200 instances - these are the regular firing model neurons. Type *B* (magenta) and *C* (cyan) neurons, belong almost entirely to the third, stuttering phenotype. Interestingly, only 26 instances, 11% of all labeled models are associated with the second phenotype, i.e. the delayed firing model. It is worth noting that the distribution of labels from our visual assessment is far from random, suggesting that the subjective features we detected in the firing patterns are, to some degree, cell type specific. It is therefore not expected that supervised classification methods, based on the training set we provide here, can accurately separate the three biophyisically different groups of neurons. Indeed, the discrimination analysis classifies the majority of the first 200 model instances (regular firing models) as type *A* (Fig. 6I,J). Stuttering type model neurons, occupying the range from 401 to 600 are classified as either type *B* or *C*. Models between 201 and 400 are identified as either type *A* or *B*. Hence, the MDA algorithm does a good job reproducing the three classes (type *A*, *B* and *C*) based on our qualitative assessment of the raster plots, but these are only vaguely associated with the biophysical phenotypes. The results of the J48 algorithm are shown in Fig. 6K,L. The output from both algorithms suggest a classification scheme that is based on qualitative temporal features of the neurons’ firing pattern rather than their biophysical properties.

### Classification based on qualitative features agrees with clustering of ISI-based vectors

**Figure 7:**
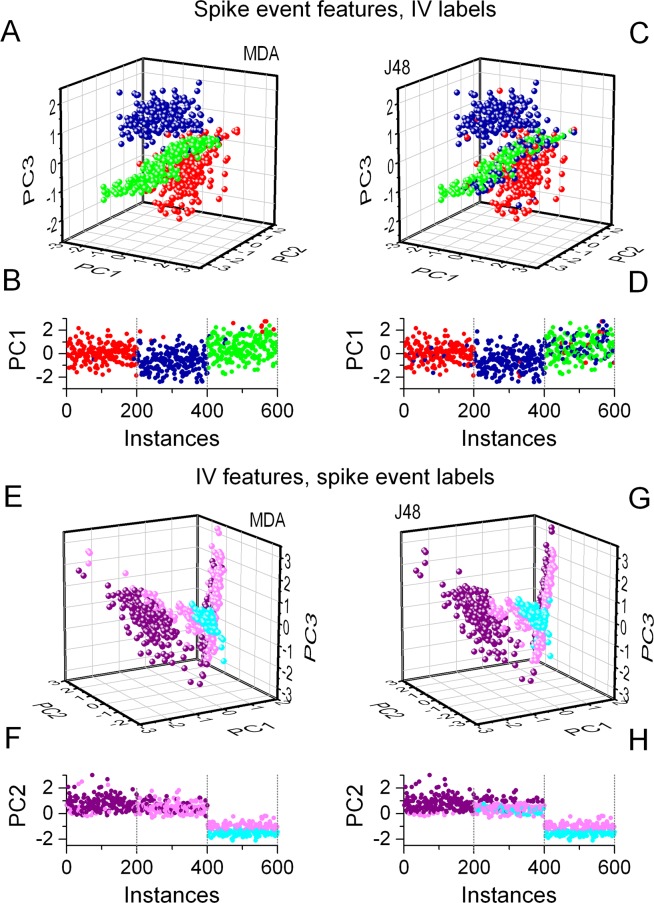
Supervised classification algorithms of model cells based on their spike event reliability perform well when labels from the IV responses are used as training set. Here, the MDA and J48 methods classify the model cells into 3 clearly separate classes (**A,C**) very well reproducing the distribution of the biophysical phenotypes (**B,D**). Conversely, when the training dataset from the spike event data is used to classify the IV feature vectors, the two methods identifies groups that represent the qualitatively different type *A*, *B* and *C* firing patterns. (**E**–**F**) MDA method, (**G**–**D**) J48.

Nevertheless, the two supervised methods can still identify the three groups of the biophysical phenotypes when spike event reliability vectors are labeled according to the training set we created for the IV feature vectors (i.e. the IV labels). Figure 7 shows that the MDA algorithm correctly classifies the vast majority of data and reproduces the distribution of the regular firing, delayed firing and stuttering types models (Fig. 7B) when using these IV labels. The decision tree method also works well, however, the number of model cells classified as delayed firing types (blue) is slightly excessive here (Fig. 7D). As the reverse approach, we can use the training set from the spike event data (labels *A*, *B* and *C*) to classify the IV feature vectors. As shown above, the supervised methods worked excellently when using the ‘right’ type of training set for the IV data, but we would not anticipate that the MDA and J48 methods reproduce the correct distribution of the 3 biophysical phenotypes when using the ‘wrong’ training set. Indeed, these two methods reproduce the ‘subjective’ distribution of model cells according to the qualitative features of their firing instead of their biophysics (Fig. 7F,H). Considering the consistency of class membership distributions both from the IV and spike event datasets we suggest that the classification using the *A-B-C* labels represents a valid interpretation of data even though this scheme does not match the 3 biophysical phenotypes.

Class membership data from the clustering of ISI-based vectors (Fig. 5B,D) and the supervised classification of spike event reliability vectors (Fig. 6J,L) were in good agreement. The best fit is found between the k-means data from the ISI-based vectors and the MDA data from the spike event reliability vectors, with 83.2% of class membership values matching. Hence, type *A*, *B* and *C* neurons, as labeled by our subjective evaluation of spike raster plots, largely overlap with the three groups of neurons that are clustered by their ISI-based parameters. This observation further supports the notion that clustering of ISI-based feature vectors offers a valid classification scheme. In this case functionally similar neurons are grouped together, but their biophysical characteristics can be quite different.

### Spike events that discriminate among biophysically different model neurons

**Figure 8:**
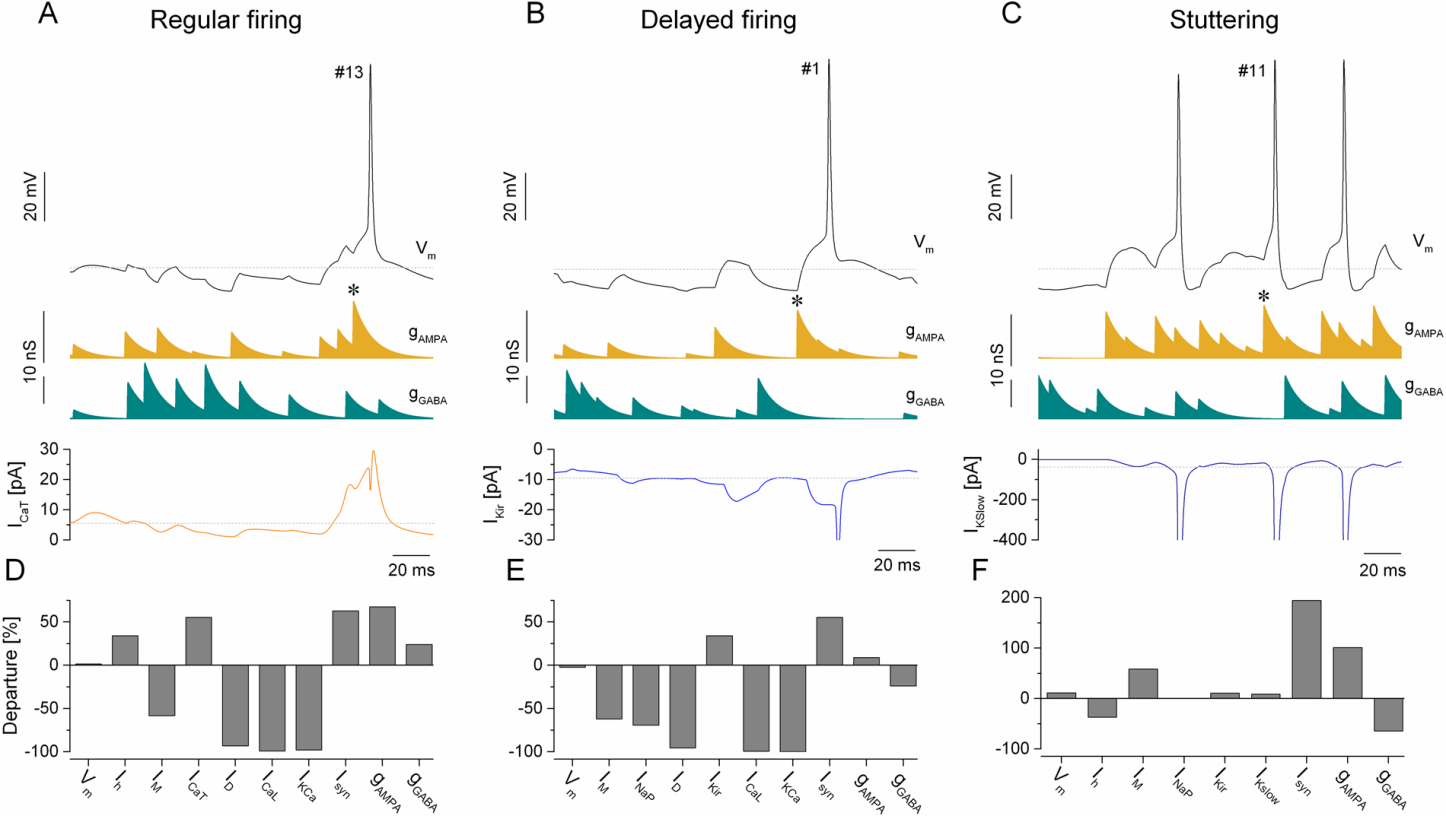
Analysis of the membrane potential history preceding the discriminating spike events explains the emergence of cell type specific firing responses. (**A**) The membrane potential of the regular firing model (black), the AMPA- (gold) and GABA-conductance (cyan) waveforms and the progression of the intrinsic Ca_T_-current (orange) are displayed before the discriminating spike event #13. (**B,C**) are similar plots for the delayed firing and stuttering models, respectively. The bottom panels **D**, **E** and **F** display the departure of the membrane potential, the intrinsic voltage-gated currents, the synaptic conductances and the net current from their respective means.

The finding that biophysical properties of neurons are to some degree ‘encoded’ in the spike event reliability vectors motivates further analysis. One can assume that reliability values at certain spike event locations can be markedly different for the three groups of biophysically different neurons (Fig. S2). To identify such presumed discriminating spike events, we utilized the simple one-rule based classification method by Holte^22^. Next, we analyzed the dynamical behavior of the membrane potential and synaptic conductance waveforms preceding the discriminating spike event. Our goal was to explain, at least qualitatively, how the intrinsic biophysical properties of the model neurons shaped their firing behavior near the locations of the most discriminating spike events. For the regular firing type neurons, spike event #13 was found as the most discriminating one. Based on the one-rule method, 84% of regular firing type neurons were correctly identified at this event location. Examining the synaptic input preceding the spike event we notice strong inhibitory conductance transients followed by excitation (Fig. 8A). In effect, this drives the membrane potential to a more hyperpolarized level and promotes the activation of the intrinsic T-type Ca-current. To quantify this effect, we calculated the mean T-current level for the entire duration of the stimulus sweep and the exponentially weighted average of the same current preceding the spike emission. The departure value was calculated as the ratio between these two means. According to this analysis the T-current becomes dominant before the spike emission exhibiting a + 55% departure from the mean (Fig. 8D). Also, the h-current is more activated (by 34%) than the mean level contributing to additional depolarization of the membrane. Indeed, the event reliability at this particular location averaged across all 200 regular firing models is 44.0 ± 7.2% (mean ± S.D.), which is 1.69 and 6.03-times greater than those values for the delayed and stuttering type models, respectively.

Turning our attention to the delayed firing model neurons, we find a different mechanism that selects the most discriminating spike events. Here, the one-rule method identifies the first spike event location (event #1, 80% accuracy) as being the most discriminating one (Fig. 8B). Apparently, this would be shortly after the beginning of the stimulus sweep. This observation suggests that the behavior of the delayed firing type neurons during the transition from rest to firing mode is markedly different from that of the other two types. We recognize that the strong intrinsic K_ir_-current in such model neurons keeps the resting membrane potential at a more hyperpolarized level than in the regular or stuttering type neurons. Indeed, the inward rectifying current has to be sufficiently deactivated before the neuron can fire. Accordingly, the spike event reliability at location #1 should be lower in this phenotype than in the other two. This is exactly what we observe: an average of 29.0 ± 11.8% spiking reliability for the delayed firing type vs. 48.2 ± 8.2% and 54.1 ± 7.5% for the regular and stuttering type models, respectively. Besides, the weighted average of the K_ir_-current preceding the spike emission at event #1 is 34% higher in magnitude than the mean level during the entire sweep (Fig. 8E).

The location of the most discriminating spike event for the stuttering type neuron model is #11. In average, stuttering type model neurons fire with 69.6 ± 3.3% reliability at this location, exceeding the 56.7 ± 5.9 and 54.3 ± 5.7% values we find for the regular and delayed firing type models, respectively. Examining the synaptic input waveforms in the vicinity of this event we see higher than average excitation and weaker inhibition (Fig. 8C,F). At the same time, the membrane potential of the stuttering type neuron exhibits strong spike afterhyperpolarizations due to the action of the slowly activating K-current that is a dominant feature of this cell type (also clearly shown in Fig. 1C). One significant effect of this strong AHP is that the cell has a relatively short refractory period and interspike intervals (i.e. firing rate) tend to be reasonably stable during maintained depolarization. In effect, this will facilitate firing at low gamma-frequencies in this type of model cells. While the intrinsic M-current is also moderately increased before the event #11, that current has a lower effect in regulating the firing rate of than the slowly activating K-current. The impact of the K_slow_-current is also clearly observed when the stuttering neuron is stimulated with static current steps: it tends to start firing at higher levels of depolarizing current, usually with single spikes or emitting short bursts. At even stronger positive current levels, the neuron fires with very stable interspike intervals during the input (Fig. 3H). Inspecting the behavior of the stuttering model near event #11 in Fig. 8C, we observe a triplet of spikes in response to stronger than average excitatory synaptic input and the interspike intervals are 53 and 38 ms. These values are, indeed, consistent with low gamma frequency firing that is a hallmark of the stuttering phenotype. Therefore, the high event reliability value we observe at this location might be due to a temporary match between the frequency of the net synaptic input and the ‘preferred’ firing frequency of the postsynaptic cell.

## Discussion

One of the motivating challenges of todays’ neuroscience is to understand the degree and role of diversity of neuronal properties in the brain^23,24^. It is therefore important to examine how consistently neuron populations are classified based on different morphological, physiological, and neurochemical criteria^7,24–26^. It has been shown that neurons with vastly different molecular properties can share similar firing characteristics^27,28^ suggesting discrepancies in the classification schemes applied to these neurons. Clustering of neurons based on their cellular physiological and synaptic properties can also yield inconsistent categorization^1^. Following this reasoning we performed our classification study on model neurons receiving different types of stimulation and demonstrated the validity of alternative classification schemes.

The feature vectors we used here serve as different representations of the neurons’ functional characteristics. The first set of data included well-established physiological parameters of neurons that are commonly obtained in whole-cell patch clamp experiments and also used in the vast majority of studies tackling the physiological diversity of neurons. We have recently shown in computational models and in dynamic clamp experiments that synaptic responses are often regulated far more potently by voltage-gated currents than static excitability^11,13^. Accordingly, one can assume that natural cell-to-cell variability in the magnitude and distribution of voltage-gated currents could impose an increasingly greater variability in the firing patterns of neurons as they respond to realistic synaptic inputs. Hence, we were motivated to check for such ‘noise amplification’ effect in our spike time data recorded under simulated synaptic bombardment. As it turned out, our initial expectation was not confirmed by the data and accurate discrimination of the 3 biophysical phenotypes was still possible using spike event reliability data.

The use of spike arrival times in our analysis is motivated by other factors, too. One has to recognize that postsynaptic neurons, in most cases, ‘read’ spike arrival times of presynaptic partners in their communication, rather than sensing the continuously evolving membrane potential of the presynaptic cells. In our model simulations we presented identical patterns of synaptic inputs to different neurons and recorded their spike arrival times. At the same time, no information on the membrane potential dynamics, or the composition of the input waveform is provided for the algorithms. It comes therefore as a surprise that unsupervised classification techniques work so well in discriminating the 3 biophysically different phenotypes. Clearly, intrinsic properties, including the voltage-gated conductances activating upon hyperpolarization, do participate in shaping the firing pattern of neurons under complex synaptic inputs. It is easy to find many examples of model neurons in our dataset that exhibit similar excitability profiles (I-O functions, Fig. 4D) even if they belong to different biophysical phenotypes. Such model neurons exhibit similar threshold of firing and slope of their I-O function, but the temporal pattern of spikes for each sweep of stimulation is slightly different. Indeed, visual observation of the entire firing pattern – the peri-stimulus raster plot – indicated neurons with distinctive integrative properties potentially belonging to different biophysical classes. Yet, as we discovered after comparing the training labels (*A-B-C*) with the original biophysical phenotype labels, our subjective assessment turned out to be inaccurate. In fact, the biophysically relevant, cell type specific differences in the firing patterns are not readily picked up by visual evaluation. Instead, small differences in the temporal order of spike appearances encode the discriminant, cell type specific features in the firing response. This is in agreement with our prior observations on 3 types of BNST neurons in dynamic clamp experiments^10^. Also, considering the superb performance of some simple phenomenological models (integrators) in predicting firing output of biological neurons under noisy stimulation^29^, one can suggest that voltage-dependent membrane currents primarily influence the fine structure rather than the gross parameters of the firing responses.

Considering the apparent mismatch between the biophysical phenotypes and the subjective *A-B-C* labels one could argue that the value of ‘expert’ assessment of spike time data is rather limited in regard to the classification of neurons. This seems especially relevant whenever electrophysiologists make assumptions on the biophysical/integrative properties of neurons by observing their activity extracellularly, such as when recording single-unit activity in active brain circuits. However, the non-random distribution of labeled model instances (Fig. 6A) suggested that our visual assessment was not entirely wrong. We recognized qualitative differences in the gross temporal features of the firing output rather than subtle differences in spike timing and order of appearance. Indeed, using ISI-parameter based vectors for unsupervised methods we obtained clusters that nicely matched those from the supervised MDA and J48 methods. Without knowing the temporal structure of the complex synaptic drive neurons receive *in vivo*, investigators often utilize firing rate, spike density functions and ISI-based temporal parameters as primary descriptors of neuronal activity^30^. At this level of analysis clustering and classification of firing responses might yield results that do not accurately reflect the underlying distribution of biophysically different cell types. It is also noteworthy that cell types identified by the ISI-based temporal features might be associated with neuron groups that share similar patterns of synaptic inputs rather than similar biophysical properties.

It is also possible that the ISI-features we used to characterize the statistical properties of the firing are more closely related to the suprathreshold behavior of neurons rather than how they function during hyperpolarization. We explored this idea by performing k-means clustering on a substantially reduced dataset of IV features. Specifically, we selected only those 4 features that were associated with the models’ behavior above spike threshold. These were the rheobase, I-O gain, the ISI coefficient of variation and the ISI accommodation parameter. Notably, k-means clustering of these 4-dimensional vectors resulted in a distribution that was very similar to the one we obtained by using the subjective *A-B-C* labels for MDA (Fig. S3). A high degree of overlap was also found between these distributions and the ones we obtained by running the k-means and HC methods on the ISI-based vectors (83.2 and 76.0%, respectively). Apparently, excluding subthreshold parameters (e.g. voltage sag) from the IV feature vectors changes the results of the k-means clustering in a way that verifies our subjective evaluation of the neurons firing responses. In this scheme, neurons are classified according to their qualitative firing properties (e.g. excitability, regularity of firing, burstiness) rather than by their biophysical composition.

In conclusion, we find that conventional current step experiments remain valuable tools in identifying and classifying biophysically different types of neurons. However, functionally, rather than biophysically different phenotypes can be revealed by classification of neuronal firing responses under complex synaptic inputs.

## Methods

### Computational models of three neuronal phenotypes

The 3-compartmental model neurons and the synaptic connections were based on the formalism described in^11^. We designed three types of model neurons each consisting of a dendritic, a somatic and an axonic compartment. Passive membrane properties and parameters of the voltage-dependent currents are listed in supporting Tables S1 and S2, respectively. All intrinsic voltage-dependent currents were implemented as standard Hodgkin-Huxley types:

$${I}_{i}={g}_{i}{m}_{i}^{p}{h}_{i}({E}_{i}-V),$$

The total maximal conductances (*g*_*i*_) of the currents for each model instance were randomly drawn from a Gaussian-distribution to simulate a biophysically diverse neuron population. The reversal potential of the currents (*E*_*i*_) was not varied across implementations. Two-hundred model instances were simulated for each biophysical phenotype. Differential equations for the activation (*m*) and inactivation (*h*) shared the same form (*x* being either *m* or *h*):

$$\frac{dx}{dt}=\frac{{x}_{\infty }(V)-x}{{\tau }_{x}(V)},$$

where voltage-dependent steady-state activation and inactivation were described by sigmoids:

$${x}_{\infty }(V)=\frac{1}{2}+\frac{1}{2}\,\tanh (\frac{V-{V}_{x,1/2}}{{V}_{x,sl}}).$$

Here, *V*_*x*,*1/2*_ denotes the midpoint of the activation/inactivation sigmoid and *V*_*x*,*sl*_ is its slope. Time constant of the activation and inactivation are bell-shaped functions of the membrane potential:

$${\tau }_{x}(V)=({\tau }_{x,max}-{\tau }_{x,min})[1-\,\tanh \,{(\frac{V-{V}_{\tau x,1/2}}{{V}_{\tau x,sl}})}^{2}]+{\tau }_{x,min}.$$

Here, *τ*_*x*,*max*_ and *τ*_*x*,*min*_ indicate the maximal and minimal levels of the bell-shaped functions, The Ca-dependent K-current and internal Ca-dynamics were based on the formalism in^31^. Synaptic currents were described using a first-order kinetics of transmitter release^32^ as:

$${I}_{syn}={g}_{syn}S({E}_{syn}-V),$$

where *S* is the instantaneous synaptic activation term yielding the following differential equation:

$$\frac{dS}{dt}=\frac{{S}_{\infty }({V}_{pre})-S}{{\tau }_{syn}(1-{S}_{\infty }({V}_{pre}))}.$$

The steady-date synaptic activation term depends on the presynaptic membrane potential as

$${S}_{\infty }({V}_{pre})=\,\tanh (\frac{{V}_{pre}-{V}_{th}}{{V}_{slope}}),$$

when *V*_*pre*_ > *V*_*th*_, otherwise ${S}_{\infty }({V}_{pre})=0$. *V*_*pre*_ denotes the presynaptic membrane potential waveform that is stored in ASCII files and designed prior to the model runs^11^. The reversal potential of the excitatory and inhibitory synaptic connections was 0 and −72 mV, respectively.

### Static features extracted from current step responses

Voltage responses of the model neurons were elicited by using stimulation with rectangular current steps. Gradually more depolarizing current steps of 400 ms duration were applied in the somatic compartment to obtain a family of voltage traces for each model instance. By analyzing these voltage responses, we extracted a total of 38 physiological parameters such as resting membrane potential, input resistance and others. Among the 38, there were parameters that were strongly correlated and redundant, hence detailed characterization of the physiological properties of neurons was possible by using a smaller number of features. We selected 20 descriptive parameters (IV features) serving as input data for the subsequent multivariate analysis. Here we aimed to identify parameters that were largely uncorrelated and also informative in distinguishing between neurons with distinct physiological character. The majority of the 20 physiological parameters were calculated from subthreshold voltage traces. Morphological features of action potentials proved to be of less value in differentiating between neuronal phenotypes, because those parameters were quite similar across model instances, therefore, we did not include those in the multivariate analysis. The description of the physiological parameters is shown in Table 1.

### Spike event reliability vectors

The second type of stimulation was designed to elicit firing responses under simulated synaptic inputs. Concurrent excitation and inhibition via AMPA- and GABA-type synaptic connections were set up using independent voltage waveforms to simulate input from excitatory and inhibitory presynaptic neurons (Fig. 2A, red and teal traces). The voltage waveforms consisted of 5 ms wide spike-shaped voltage transients that departed from and returned to a rest state of −60 mV. In order to induce variable amplitude synaptic currents in the postsynaptic model neuron, we imposed amplitude variation of the spike-shaped voltage transients such that their peak value ranged from −30 to 0 mV in a uniform distribution^13^. Mean firing rate of the waveforms was 30 Hz and interspike intervals were drawn from a Poissonian distribution. The duration of the synaptic conductance waveforms was 8 s and the strength of the AMPA- and GABA-connection was incremented by 0.25 and 0.5 nS, respectively, in the successive repetitions of stimulation. Altogether, 101 of such sweeps were presented to elicit gradually more intense firing and yielding spike event raster diagrams such as the one in Fig. 4E. We note that such stimulation shares similarities with the static current step stimulation we used to calculate the static physiological parameters. By gradually increasing the strength of the excitatory synaptic drive from 0 to 25 nS we elicited an increasing number of action potentials. Analogously to the static I-O relationship, a “dynamic input-output function” can be obtained by plotting spike counts against the AMPA-conductance (Fig. 4D). The window width we used to determine event reliability values was 33 ms. More precisely, a postsynaptic spike was associated with a presynaptic spike whenever it was emitted at least 2 ms after the presynaptic spike but not later than 35 ms. Spike event reliability in 52 locations was determined yielding 52-dimensional vectors for the subsequent principal component analysis and clustering.

### Multivariate analysis

#### Principal component analysis

Prior to running clustering algorithms on electrophysiological parameters and spike event reliability we performed principal component analysis (PCA). This technique can be used to reduce the dimensionality (number of parameters) of a multiparameter dataset by generating new uncorrelated variables^33^. PCA applies a multi exponential fit to the data, and each new uncorrelated variable (principal component, PC) can describe more than one original parameter. We performed PCA with standardized scores using the data analysis/graphing software Origin 2016. PCA is most useful when it can identify two or three PCs to describe most of the variance in a dataset. In our case this condition was met, so we used 3-dimensional PCA-transformed data for the subsequent clustering and classification.

#### K-means clustering

K-means is a clustering algorithm in which points are iteratively reassigned to clusters in to minimize within-cluster distances. We used k-means clustering in Weka 3.8 (University of Waikato, Hamilton, New Zealand). The clustering was performed using Euclidean distance metric. Ten starting seeds were initialized with random assignment of all cells to 3 groups and 500 maximum iterations.

#### Hierarchical clustering

This clustering method begins by separating each individual neuron into a cluster by itself. At each stage of the analysis, the neurons that are most similar, as characterized by our electrophysiological features, are grouped together to form another larger cluster. This process continues until all of the neurons are joined into a single cluster. Ward’s method^34^ minimizes the error sum of squares of any pair of clusters formed at a given step; this maximizes between-group differences and minimizes within-group differences. Hierarchical cluster analysis based on Ward’s method and using squared Euclidian distance was performed in Origin to classify the 3 cell types. The variables were not standardized.

#### Multidiscriminant analysis

Discriminant analysis was performed in SPSS. This is a classification method that uses a training set (instances labeled by the person performing the analysis) to train a classifier and predicts the class membership of unlabeled instances. The stepwise method was performed with Mahalonobis distance and the prior probabilities were computed from group sizes.

#### J48 decision tree

J48 is a classifier for generating a pruned or unpruned C4.5 decision tree as described^35^. At each node of the tree, C4.5 chooses the attribute of the data that most effectively splits its set of samples into subsets enriched in one class or the other. The splitting criterion is the normalized information gain (difference in entropy). The attribute with the highest normalized information gain is chosen to make the decision. The J48 method was ran in Weka 3.8. One starting seed was initialized with random assignment and confident factor of 0.25.

#### One-rule based classification

We used the one-rule method to find spike event locations that best discriminated a selected model phenotype. For each phenotype we determined the event location (one of the 52 event locations) where the best separation was achieved by using a single cut-off value on the spike event reliability scale.

### Analysis of the membrane potential waveforms

To characterize the impact of various dynamical variables in the close proximity of selected spike events, we calculated their departure from their corresponding total arithmetic mean values. We used exponential weighting of the membrane potential (*V*_*m*_) and the various voltage-gated current waveforms and calculated their weighted averages using samples preceding the spike event of interest. The time constant of the weight function was set as twice the membrane time constant of the neuron model determined from the corresponding voltage responses under negative current steps (as in Fig. 1A–C). Non-weighted averages of all variables were calculated for the entire duration of the stimulus sweep as arithmetic means and the departure values were calculated as the ratio of the weighted and non-weighted means.

## Supplementary information

Supplementary information
