# Supplementary material for: Alternative classifications of neurons based on physiological properties and synaptic responses, a computational study
Source: Sci Rep. 2019 Sep 11;9:13096. doi: 10.1038/s41598-019-49197-8 (PMC6739481; doi:10.1038/s41598-019-49197-8)
Supplement: Supplementary file 1 — Supplementary information [file 41598_2019_49197_MOESM1_ESM.pdf]

Alternative classifications of neurons based on physiological properties and synaptic responses, a computational study

Ferenc Hernáth[1], Katalin Schlett[1] and Attila Szücs[1,2,3 *]

[1] MTA-ELTE-NAP B Neuronal Cell Biology Research Group, Eötvös Loránd University, Budapest, Hungary

[2] BioCircuits Institute, University of California San Diego, La Jolla, California, United States

[3] Balaton Limnological Institute of the Center for Ecological Research, Hungarian Academy of Sciences, Tihany, Hungary

* Corresponding author, E-mail: aszucs@ucsd.edu

Supplementary information

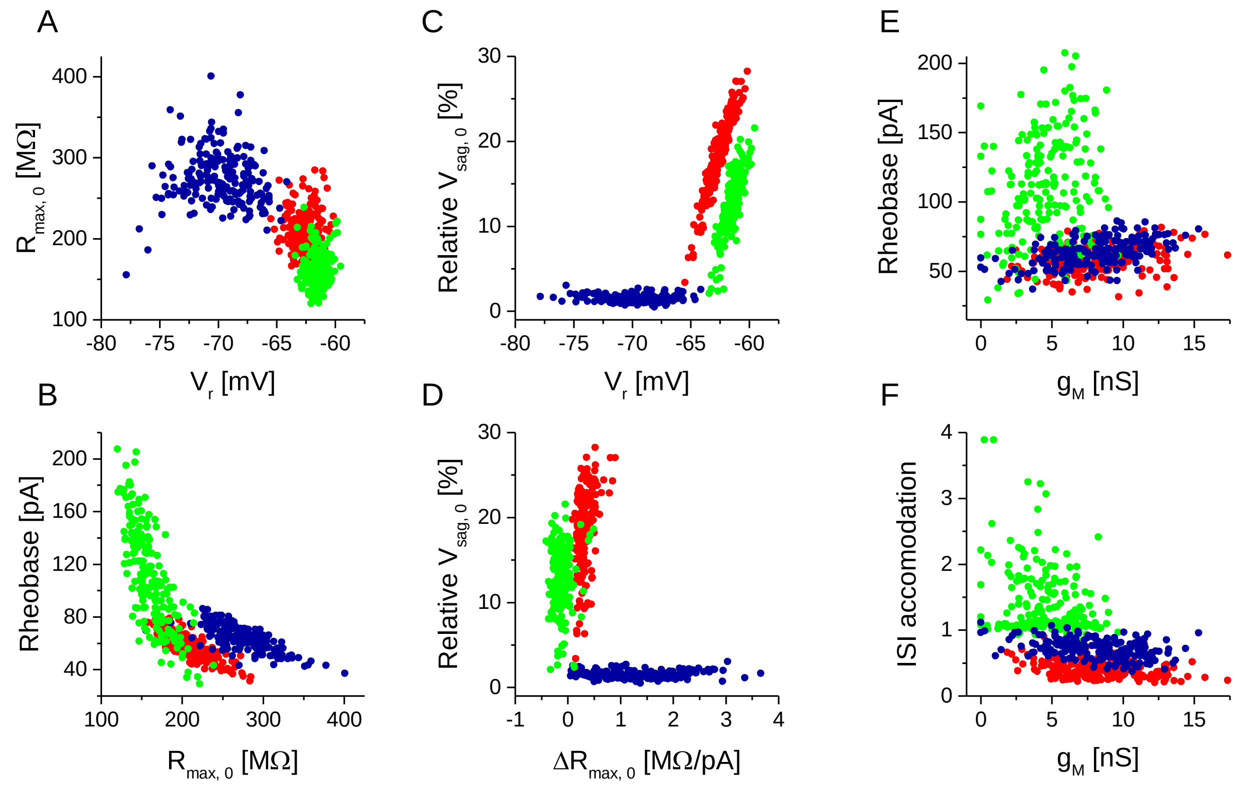

Fig S1. Physiological parameters extracted from voltage responses of the model neurons exhibit variable degree of overlap across the 3 phenotypes. In panels A-D one selected physiological parameter is scatter plotted against another. Red, blue and green points indicate data from the regular firing, delayed firing and stuttering type model neurons, respectively. In E the rheobase of the model neurons is plotted against the maximal conductance of the intrinsic M-current. F shows the ISI accommodation vs. M-current conductance relationship.

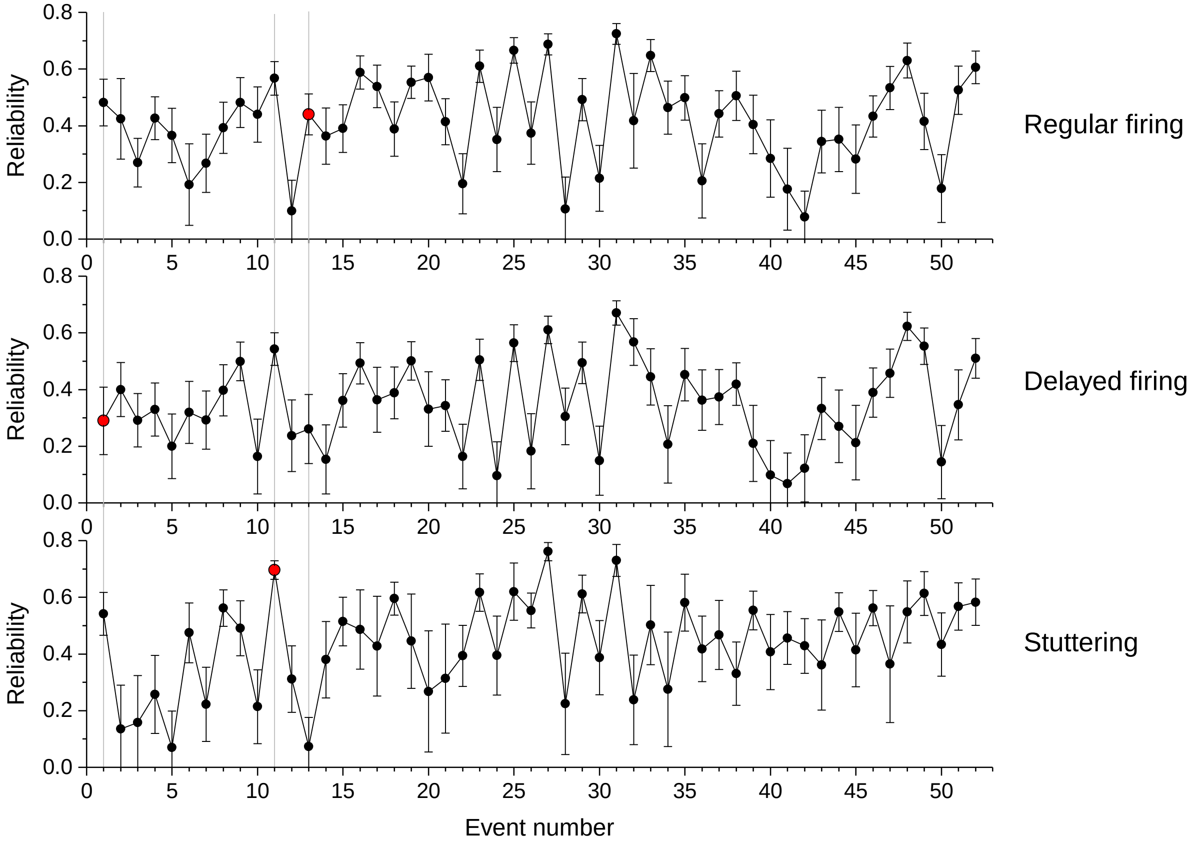

Fig S2. Spike event reliability values are shown for the 52 even locations and for the three types of model neurons. Error bars indicate standard deviations (n=200 for each phenotype). The most discriminating event locations are highlighted with red symbols (#13 for the regular firing; #1 for the delayed firing and #11 for the stuttering type models).

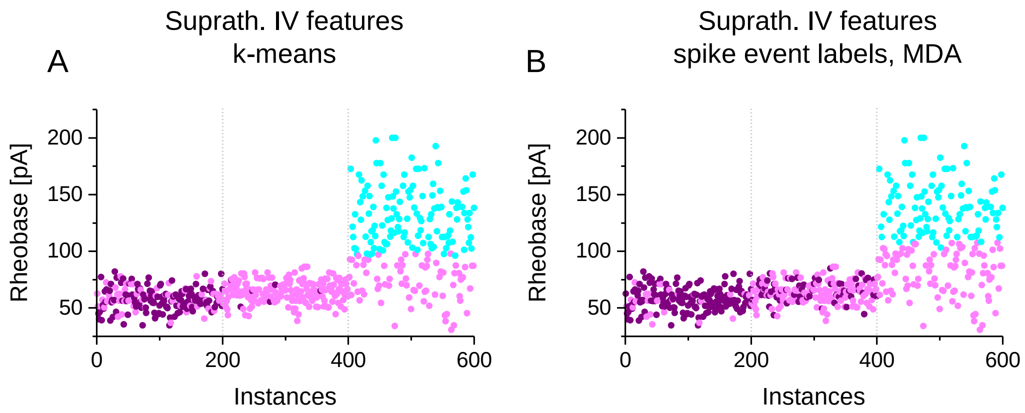

Fig S3. Class membership distributions from k-means clustering of reduced 4-dimensional IV data vectors and from discriminant analysis of the same data are remarkably similar. In panel A the rheobase of the model neurons is plotted against their instance number and symbols are colored according to the class membership determined by the k-means algorithm. Here, no PCA was performed prior to clustering. B shows the output of the MDA method when the suprathreshold IV features were used as input and the training set was as shown in Fig 5B. The match between the two class membership distributions is 80.2%.

	Soma		Axon		Dendrite				
	g_{leak} nS	C_m pF	g_{leak} nS	C_m pF	g_{leak} nS	C_m pF	g_{sx} nS	g_{sd} nS	E_{leak} mV
Regular	2.0	60	1.5	20	1.0	10	18	16	-66.0
Delayed	2.0	60	1.5	20	1.0	10	18	16	-64.0
Stuttering	2.5	40	1.5	15	1.0	10	20	16	-62.0

Table S1. Passive membrane parameters of the three neuron models. g_{sx} is the electrical coupling conductance between the soma and axon compartments. g_{sd} indicates the coupling between the soma and the dendrite compartments.

Curr	Typ	g nS	E mV	So %	Ax %	De %	p	$V_{m,1/2}$ mV	$V_{m,sl}$ mV	$V_{h,1/2}$ mV	$V_{h,sl}$ mV	$\tau_{m,max}$ ms	$\tau_{m,min}$ ms	$V_{tm,1/2}$ mV	$V_{tm,sl}$ mV	$\tau_{h,max}$ ms	$\tau_{h,min}$ ms	$V_{th,1/2}$ mV	$V_{th,sl}$ mV
Na	R<br>D<br>S	8000<br>9000<br>9000	55	25	75	0	3	-27	14	-54	-14	0.9	0.1	-68	30	8	8	5	30
Na_P	R<br>D<br>S	-<br>1.0<br>2.0	50	33	0	67	1	-26	12			3.0	0.2	-70	50				
H	R<br>D<br>S	3.0<br>-<br>3.0	-40	33	0	67	1	-73	-16			200	15.0	-62	30				
K_d	R<br>D<br>S	400<br>400<br>250	-72	25	75	0	4	-22	15			10	0.7	-70	30				
M	R<br>D<br>S	8.0<br>8.0<br>5.0	-75	50	50	0	1	-28	17			80	20.0	-80	100				
D	R<br>D<br>S	200<br>150<br>-	-80	50	50	0	3	-31	20	-73	-15	2.5	0.2	-50	30	100	100	-75	50
K_{ir}	R<br>D<br>S	-<br>8.0<br>2.0	-85	67	0	33	1	-80	-15			50	3.0	-45	40				
K_{slow}	R<br>D<br>S	-<br>-<br>300	-75	50	50	0	1	-26	12	-59	-13	6	6	-60	30	500	50	-25	-30
Ca_T	R<br>D<br>S	25<br>-<br>-	90	100	0	0	2	-49	14	-83	-13	5.0	0.8	-68	30	50	5	-75	25
Ca_L	R<br>D<br>S	20<br>10<br>-	90	50	0	50	2	-23	13	-53	-13	10	1.0	-54	27	50	5	-65	27

Table S2. Parameters of the voltage-dependent currents for the three neuronal phenotypes (R: regular firing, D: delayed firing, S: stuttering). So, Ax and De indicate the percentage of conductance allocated for the soma, axon and dendrite compartments.
